# Notification that new names of prokaryotes, new combinations and new taxonomic opinions have appeared in volume 76, part 3 of the IJSEM

**DOI:** 10.1099/ijsem.0.007148

**Published:** 2026-06-30

**Authors:** Aharon Oren, Markus Göker

**Affiliations:** 1 The Institute of Life Sciences, The Hebrew University of Jerusalem, The Edmond J. Safra Campus, 9190401 Jerusalem, Israel; 2 Leibniz Institute DSMZ – German Collection of Microorganisms and Cell Cultures, Inhoffenstrasse 7B, 38124 Braunschweig, Germany

This listing of names of prokaryotes published in a previous issue of the International Journal of Systematic and Evolutionary Microbiology (IJSEM) is provided as a service to microbiology to assist in the recognition of new names and new combinations. This procedure was proposed by the Judicial Commission [1]. The names given herein are listed according to the rules of priority (i.e. time and date of publication of the articles on the journal’s platform and the order of valid publication of names in the original articles) [2].

**Table:** 

Order of article publication	Name/author	Proposed as	Article no.	Page no.
1	*Mannheimia kashgarensis* Yang *et al*. 2026	sp. nov.	007089	5
2	*Methanothermococcus jasoni* Rizzo *et al*. 2026	sp. nov.	007098	5
3	*Bartonella bennetti* Brierley *et al*. 2026	sp. nov.	007095	10
4	*Microvirga medicaginis* Zheng *et al*. 2026	sp. nov.	007058	6
5	*Gardnerella lacydonensis* Allini Ntiguemassa *et al*. 2026	sp. nov.	007028	16
5	*Gardnerella bretellae* Allini Ntiguemassa *et al*. 2026	sp. nov.	007028	16
5	*Gardnerella massiliensis* Allini Ntiguemassa *et al*. 2026	sp. nov.	007028	16
5	*Gardnerella phocaeensis* Allini Ntiguemassa *et al*. 2026	sp. nov.	007028	17
6	*Methylobacterium endophyticum* Jiang *et al*. 2026	sp. nov.	007090	7
6	*Endoryza* Jiang *et al*. 2026	gen. nov.	007090	7
6	*Endoryza foliicola* Jiang *et al*. 2026	sp. nov.	007090	8
7	*Actomonas mangrovi* Wang *et al*. 2026	sp. nov.	007101	8
7	*Actomonas spartinae* Wang *et al*. 2026	sp. nov.	007101	8
7	*Actomonas lomoniglobus* (Ahmad *et al*. 2024) Wang *et al*. 2026	comb. nov. [basonym: *Synoicihabitans lomoniglobus* Ahmad *et al*. 2024]	007101	8
8	*Homoserinimonas flava* Luo *et al*. 2026	sp. nov.	007085	10
9	*Limosilactobacillus secundus* Guo *et al*. 2026	sp. nov.	007099	7
9	*Limosilactobacillus reuteri* subsp. *pararodentium* Guo *et al*. 2026	subsp. nov.	007099	7
9	*Limosilactobacillus reuteri* subsp. *peregrinus* Guo *et al*. 2026	subsp. nov.	007099	8
9	*Limosilactobacillus reuteri* subsp. *simiae* Guo *et al*. 2026	subsp. nov.	007099	8
10	*Sphingobium wonjuense* Jeong and Kim 2026	sp. nov.	007100	8
10	*Sphingobium erigeronicola* Jeong and Kim 2026	sp. nov.	007100	8
11	*Sphingomonas lindongxini* Cao *et al*. 2026	sp. nov.	007094	8
11	*Sphingosinicella wutangchuni* Cao *et al*. 2026	sp. nov.	007094	9
12	*Acidaminococcus nyctereutis* Li *et al*. 2026	sp. nov.	007103	7
13	*Arboribacterium* Kobayashi *et al*. 2026	gen. nov.	007087	9
13	*Arboribacterium acericorticis* Kobayashi *et al*. 2026	sp. nov.	007087	9
14	*Ruicaihuangia ninglii* Luo *et al*. 2026	sp. nov.	007096	8
14	*Ruicaihuangia jiafui* Luo *et al*. 2026	sp. nov.	007096	9
14	*Homoserinibacter chengtaonis* Luo *et al*. 2026	sp. nov.	007096	9
15	*Janibacter zhaoxuefangae* Zhang *et al*. 2026	sp. nov.	007097	7
15	*Janibacter zhangyanbonis* Zhang *et al*. 2026	sp. nov.	007097	8
16	*Variovorax glucosi* Chen *et al*. 2026	sp. nov.	007105	8
16	*Variovorax frigidus* Chen *et al*. 2026	sp. nov.	007105	8
16	*Variovorax trehalosi* Chen *et al*. 2026	sp. nov.	007105	8
16	*Variovorax restrictus* Chen *et al*. 2026	sp. nov.	007105	9
16	*Variovorax cryoconiti* Chen *et al*. 2026	sp. nov.	007105	9
16	*Variovorax pasteuri* Chen *et al*. 2026	sp. nov.	007105	10
16	*Variovorax nivicola* Chen *et al*. 2026	sp. nov.	007105	10
16	*Variovorax multivorans* Chen *et al*. 2026	sp. nov.	007105	10
16	*Variovorax nitratireducens* Chen *et al*. 2026	sp. nov.	007105	11
16	*Variovorax monticola* Chen *et al*. 2026	sp. nov.	007105	11
16	*Variovorax raffinosi* Chen *et al*. 2026	sp. nov.	007105	12
16	*Variovorax glacialis* Chen *et al*. 2026	sp. nov.	007105	12
16	*Variovorax sucrosi* Chen *et al*. 2026	sp. nov.	007105	12
17	*Dolomitihabitans* Robertson and Meyers 2026	gen. nov.	007111	9
17	*Dolomitihabitans soli* (Yin *et al*. 2025) Robertson and Meyers 2026	comb. nov. [basonym: *Streptosporangium soli* Yin *et al*. 2025]	007111	9
18	‘*Candidatus* Nostocimimus’ Crook 2026*	gen. nov. [replacement name for *Candidatus* Nostocoides’ corrig. Kulichevskaya *et al*. 2012]	007115	2
18	‘*Candidatus* Nostocimimus acidiphilus’ Crook 2026*	comb. nov. [pro-basonym: ‘*Candidatus* Nostocoides acidiphilum’ corrig. Kulichevskaya *et al*. 2012]	007115	2
19	*Vibrio glycogenivorans* Tong *et al*. 2026	sp. nov.	007106	9
20	*Plantibacter lichenum* Chen *et al*. 2026	sp. nov.	007110	6
21	*Corynebacterium drakensteinense* Haines *et al*. 2026†	sp. nov.	007118	1

*The name is not pro-validly published because the nomenclatural type of the species was not designated.

†Corrigendum to Haines *et al*. 2026, IJSEM 2026;76:007068 where the name *Corynebacterium drakensteinense* was effectively but not validly published.

## Abbreviations

IJSEM: International Journal of Systematic and Evolutionary Microbiology
